# Genetic Affinities of the Central Indian Tribal Populations

**DOI:** 10.1371/journal.pone.0032546

**Published:** 2012-02-29

**Authors:** Gunjan Sharma, Rakesh Tamang, Ruchira Chaudhary, Vipin Kumar Singh, Anish M. Shah, Sharath Anugula, Deepa Selvi Rani, Alla G. Reddy, Muthukrishnan Eaaswarkhanth, Gyaneshwer Chaubey, Lalji Singh, Kumarasamy Thangaraj

**Affiliations:** 1 Department of Zoology, Government Motilal Vigyan Mahavidyalaya, Bhopal, India; 2 CSIR-Centre for Cellular and Molecular Biology, Hyderabad, India; 3 Department of Evolutionary Biology, Institute of Molecular and Cell Biology, University of Tartu and Estonian Biocentre, Tartu, Estonia; 4 Genome Foundation, Hyderabad, India; 5 Banaras Hindu University, Varanasi, India; University of Cambridge, United Kingdom

## Abstract

**Background:**

The central Indian state Madhya Pradesh is often called as *‘heart of India’* and has always been an important region functioning as a trinexus belt for three major language families (Indo-European, Dravidian and Austroasiatic). There are less detailed genetic studies on the populations inhabited in this region. Therefore, this study is an attempt for extensive characterization of genetic ancestries of three tribal populations, namely; Bharia, Bhil and Sahariya, inhabiting this region using haploid and diploid DNA markers.

**Methodology/Principal Findings:**

Mitochondrial DNA analysis showed high diversity, including some of the older sublineages of M haplogroup and prominent R lineages in all the three tribes. Y-chromosomal biallelic markers revealed high frequency of Austroasiatic-specific M95-O2a haplogroup in Bharia and Sahariya, M82-H1a in Bhil and M17-R1a in Bhil and Sahariya. The results obtained by haploid as well as diploid genetic markers revealed strong genetic affinity of Bharia (a Dravidian speaking tribe) with the Austroasiatic (Munda) group. The gene flow from Austroasiatic group is further confirmed by their Y-STRs haplotype sharing analysis, where we determined their founder haplotype from the North Munda speaking tribe, while, autosomal analysis was largely in concordant with the haploid DNA results.

**Conclusions/Significance:**

Bhil exhibited largely Indo-European specific ancestry, while Sahariya and Bharia showed admixed genetic package of Indo-European and Austroasiatic populations. Hence, in a landscape like India, linguistic label doesn't unequivocally follow the genetic footprints.

## Introduction

Indian populations are known for their unique cultural and linguistic diversity [1]. Broadly, Indian population can be categorized as the castes, tribes and religious communities. Tribes represent ∼8.2% of the total population of India [2]. There are currently about 530 tribal groups in India [2]. They vary in size from a few hundred to a few million and speak four major language families belonging to Austroasiatic (AA), Dravidian (DRA), Indo-European (IE) and Tibeto-Burman (TB). The origin of the tribal population has always been a matter of debate among the anthropologists and historians. Recent genetic studies have highlighted that the deep rooted Indian haplogroups are present everywhere, irrespective of the caste, tribe or language differentiation [1], [3].

Madhya Pradesh (MP) is the second largest Indian state by area, is located in the central part and is homeland of several caste and tribal groups. It is bordered by the states of Uttar Pradesh in the north, Chhattisgarh in the east, Maharashtra in south, Gujarat in the west and Rajasthan in the northwest. Except for the valleys of the Narmada and the Tapti, Madhya Pradesh consists of a plateau, straddled by the river Narmada and interspersed with the mountains of the Vindhya and the Satpura ranges. It is one of the largest states of India inhabited by the bulk of tribal populations of the country constituting 20.3% of the total tribal populations. There are 46 Scheduled Tribes (ST) [2], among which Gond, Bhil, Baiga, Sahariya, Oraon, Korku and Kol are the most prominent. Scheduled Tribes are communities in India which are given a special status by the Constitution of India for their development and welfare [2]. The tribal groups of MP are mainly hunter gatherers, labours and farmers and belong to IE, DRA and AA families, which are widely spread language families in India [1].

**Figure 1:**
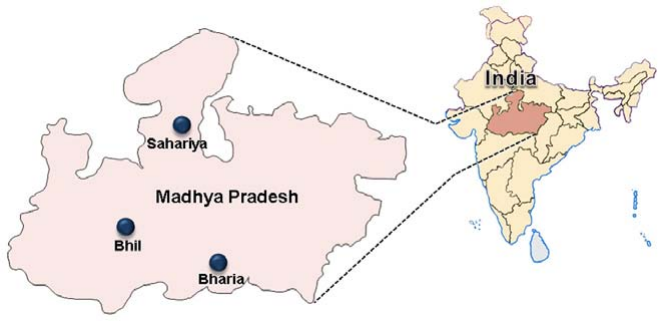
Sampling area. Map of Madhya Pradesh showing the sampling area. The samples were collected from those areas where the tribes concerned were in highly concentrated. Bhil is collected from Sehore district, Bharia from Chhindwara region and Sahariya from Shivpuri district of Madhya Pradesh.

Over the past few years, genetic studies using haploid and diploid genetic markers have provided a substantial understanding of the human origins and dispersal patterns in South Asia [4]–[8]. So far, there are scattered genetic information on the caste and tribal populations of different regions of India [3], [5], [9], [10], but the central region is less focused. As this state is a shelter for three different language groups, it can help to test several language-gene interaction models. The archaeological studies also suggest that Narmada region has played a vital role in initial peopling of the subcontinent [11], [12]. Considering its central role in shaping the major episodes of peopling of South Asia, a detailed study on the populations of this important state is necessary. Moreover, recently, it has been shown that the ancestral Indian population has given rise to many independent endogamous groups [6]. Therefore, there is a necessity to fill the big lacuna by the inclusion of this region to reveal a clear picture of the origin and genetic affinity of the Indian population in a broad context. Hence, in this high resolution study, we have made an attempt to the dissect the origin and the genetic affinities of the three tribal populations- Bharia (Dravidian), Bhil (Indo-European) and Sahariya (Indo-European) of Madhya-Pradesh state (Fig. 1 and Table S1) using haploid (Y-chromosomal and Mitochondrial DNA markers) and diploid (50 ancestry informative autosomal SNP) genetic markers.

## Results and Discussion

### Y-chromosome analysis

#### Y SNP analysis

**Figure 2:**
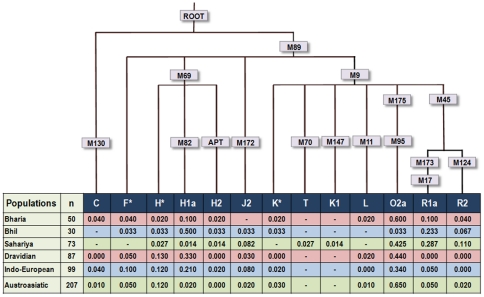
Rooted maximum parsimony tree. Rooted maximum parsimony tree of Bhil, Bharia and Sahariya tribes constructed based on Y SNP binary markers. The contemporary populations from different language groups are taken from the literature [5], [7], [9], [10], [13], [28].

We have analysed a total of 153 males with 16 Y-chromosomal biallelic markers and observed 13 haplogroups in the studied populations (Fig. 2). The frequency of haplogroup M95-O2a, which is highly frequent among Austroasiatic speakers [7], [13], was high among Bharia and Sahariya tribes (Fig. 2). The second most frequent haplogroup belonged to M207-R lineage. It has two sister clades, R1-M173 and R2-M124. The occurrence of haplogroups M17-R1a and M124-R2 was highest in Sahariya tribe, followed by Bhil and Bharia (Fig. 2). Haplogroup M82-H1a was the third most frequent haplogroup among the three studied tribal populations of this state. Its highest frequency was observed among Bhils and also with the considerable prevalence in Bharias (Fig. 2). Some of the previous studies considered M69-H haplogroup as a tribal-specific and M17-R1a haplogroup as caste-specific [14], [15]. In contrast to these studies, the present study bolsters the view that the occurrence of these haplogroups are universal in majority of populations, regardless of their caste or tribal affiliation in both caste and tribal populations of India [9]. The discrepancy of frequency distribution of these haplogroups in caste and tribal populations can be explained by their different population sizes, where evolutionary forces act in a different way and diverse social customs that involve practicing endogamy at different levels [16]. In conjugation with these frequent haplogroups, we also noticed haplogroups M130-C, M89-F*, M69-H*, apt-H2, M172-J2, M9-K*, M147-K1, and M70-T at low frequencies (Fig. 2).

#### Y-STRs analysis

**Figure 3:**
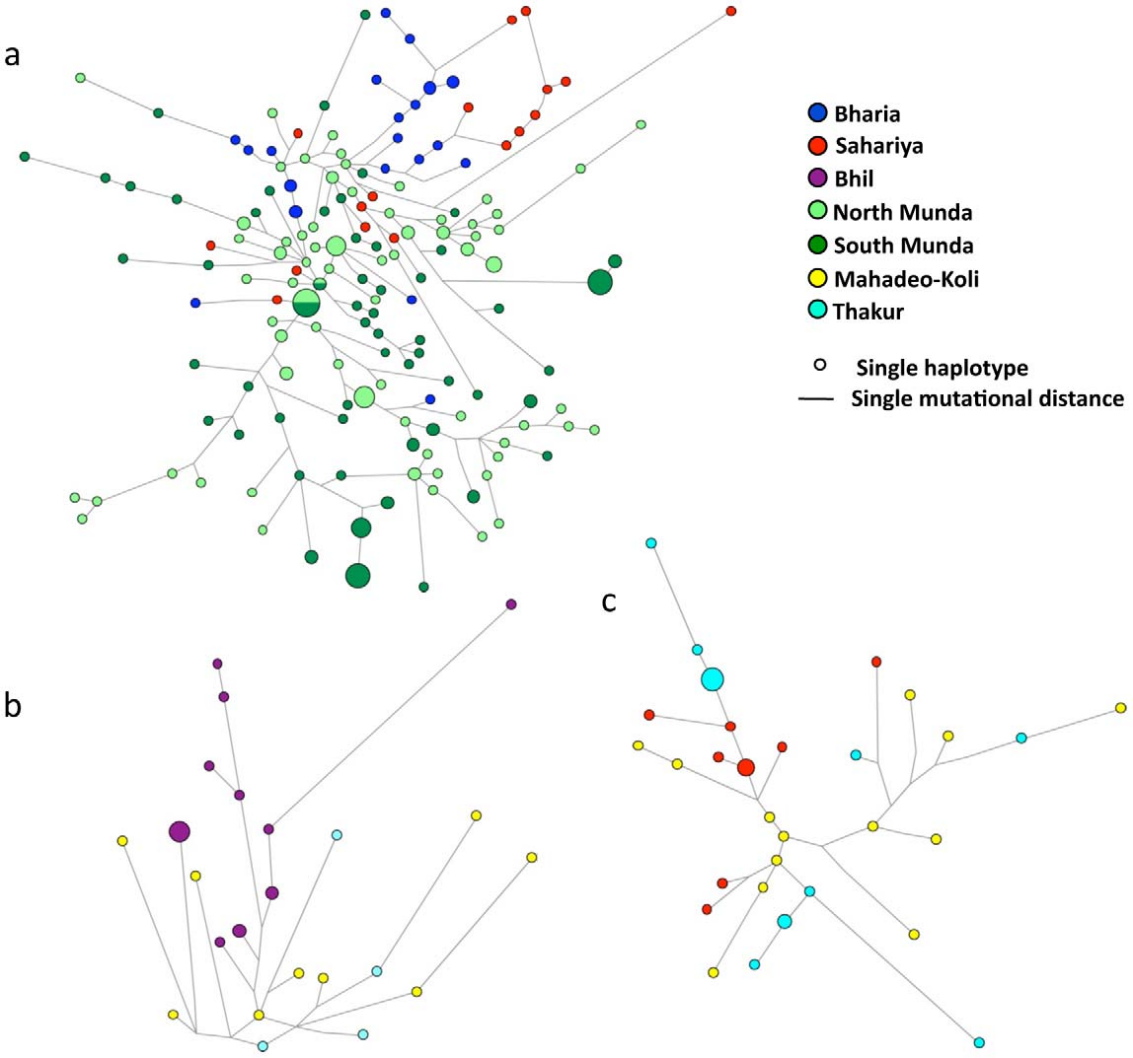
Unrooted phylogenetic networks. Unrooted phylogenetic network of a) haplogroups M95-O2a; b) haplogroup M82-H1a and c) haplogroup M17-R1a. The network was constructed using a median-joining algorithm as implemented in the Network 4.5.0.2 program. The size of the circles is proportional to the number of samples. Comparative data was taken from the literature [7], [17].

To have a deeper insight about the paternal origin of these populations, we have generated 17 Y-chromosome-specific STRs data (Table S2), in the background of their most frequent haplogroups (Fig. 2), and compared them with the published datasets [7], [17]. By using this approach, we obtained Y-STRs data from three most frequent haplogroups viz., M95-O2a, M82-H1a and M17-R1a and performed Network [18] and coalescent analysis [19]. The M95-O2a Network tree suggested a closer affinity of Bharia and Sahariya with North Munda group. Interestingly, it was seen that the founder haplotype of the Bharia tribe was branching-off from the Mawasi tribe (Fig. 3) which is indeed an AA (North Munda) tribe living in close proximity of Bharia. The founder analysis of Bharia-specific clad yielded an expansion time of 6.83±2.65 thousand years ago (kya), suggesting a geneflow from the North Munda group to Bharia. The TMRCA for O2a-M95 haplogroup in Bharia was estimated to be 13.18±3.24 kya, indicating the expansion of O2a in this region as an older event before the differentiation of any language group. Similarly, the coalescent time for M82-H1a haplogroup in Bhil and M17-R1a and M95-O2a in Sahariya was estimated to be 13.18±3.24 kya, 10.97±1.86 kya and 16.48±3.06 kya, respectively (Table 1). However, the expansion times of different lineages can be considered as the upper boundary of the migration rather than referring to the time of origin of these tribal groups. The above results suggests that the frequent Y-chromosomal haplogroups in this region are highly unlikely a newcomer or recently migrated, they are present there at least since pre-Neolithic time. From our result, it is evident that there is a dominance of different haplogroups in each of the tribal population (Fig. 2 and Table 2). This may be either due to genetic drift or due to strict endogamy practices of these populations or by both.

**Table 1:** The coalescent age of the major Y-chromosomal haplogroups observed in studied populations.

Population	n	Haplogroup	Variance	TMRCA (kya)
Bharia	24	O2a	0.29	13.18±3.24
Saharia	17	O2a	0.42	16.48±3.06
Saharia	12	R1a	0.29	10.97±1.86
Bhil	15	H1	0.45	13.18±3.24

**Table 2:** mtDNA haplogroup frequencies in the studied populations and in neighbouring groups.

Population	n	M	M2	M3	M4	M5	M6	M18	M25	M30	M31	M33	M34	M35	M36	M37	M38	M39	M40	M41	M44	M45
Bharia	65	0.015	0.000	0.046	0.000	0.031	0.077	0.031	0.000	0.000	0.000	0.062	0.000	0.031	0.077	0.000	0.000	0.015	0.046	0.062	0.000	0.108
Bhil	49	0.061	0.102	0.082	0.041	0.102	0.000	0.000	0.000	0.102	0.000	0.000	0.000	0.041	0.000	0.020	0.020	0.020	0.020	0.020	0.000	0.020
Saharia	95	0.074	0.000	0.032	0.021	0.084	0.000	0.021	0.000	0.042	0.105	0.000	0.000	0.021	0.000	0.000	0.000	0.011	0.063	0.000	0.000	0.011
Dravidian	235	0.213	0.130	0.110	0.017	0.068	0.038	0.013	0.030	0.013	0.000	0.021	0.000	0.021	0.000	0.000	0.004	0.034	0.000	0.000	0.000	0.000
Austroasiatic	175	0.046	0.051	0.040	0.046	0.149	0.046	0.023	0.011	0.006	0.023	0.006	0.006	0.017	0.000	0.000	0.000	0.046	0.150	0.006	0.000	0.091
Indo-European	65	0.154	0.138	0.062	0.015	0.108	0.000	0.015	0.000	0.000	0.000	0.000	0.000	0.000	0.000	0.000	0.000	0.000	0.015	0.000	0.015	0.015

Population	n	M4′67	M49	M53	M66	N5	W	R	R2	R5	R6	R7	R8	R31	HV	T	U	U1	U2	U7	X2
Bharia	65	0.031	0.000	0.015	0.015	0.000	0.000	0.015	0.000	0.000	0.046	0.138	0.062	0.000	0.000	0.000	0.015	0.000	0.046	0.015	0.000
Bhil	49	0.000	0.020	0.000	0.000	0.000	0.000	0.000	0.000	0.122	0.020	0.000	0.020	0.061	0.000	0.000	0.000	0.020	0.082	0.000	0.000
Saharia	95	0.011	0.000	0.000	0.000	0.232	0.000	0.011	0.011	0.000	0.084	0.032	0.000	0.000	0.000	0.000	0.000	0.000	0.063	0.011	0.063
Dravidian	235	0.000	0.000	0.000	0.000	0.000	0.009	0.089	0.000	0.051	0.009	0.004	0.000	0.000	0.013	0.009	0.000	0.000	0.081	0.009	0.000
Austroasiatic	175	0.000	0.000	0.000	0.000	0.000	0.006	0.057	0.023	0.023	0.086	0.017	0.000	0.000	0.000	0.000	0.000	0.000	0.017	0.006	0.000
Indo-European	65	0.000	0.000	0.000	0.000	0.000	0.000	0.138	0.000	0.062	0.015	0.015	0.015	0.000	0.000	0.000	0.092	0.000	0.077	0.046	0.000

Note: Comparative data is taken from the published works [3], [5], [22], [28].

### Mitochondrial DNA analysis

To determine the maternal lineages, mitochondrial DNA (mtDNA) HVS-I sequence was obtained for 209 individuals (Table S3), which were further analyzed for other diagnostic coding region and finally 20 samples were selected for complete mtDNA sequencing. Haplogroups were assigned to each individual following Global human mtDNA phylogenetic tree [20] and updated with the new information published elsewhere [7].

Consistent with other studies on Indian populations [5], [17], macrohaplogroup M was observed in majority of the individuals followed by macrohaplogroup R and macrohaplogroup U (Table 2). Apart from M* (including M4′67 paragroup) and R* (including U*), altogether, 31 haplogroups were differentiated in the studied populations (Table 2). Majority of the haplogroups observed in the present study are autochthonous to South Asia. While, none of the East Asian-specific haplogroups were seen in the studied populations (Table 2); suggesting a negligible female geneflow from East Asia to this region. Considering the recent observation that the Austroasiatic populations of India migrated from Southeast Asia to India [7] and the distribution of several Austroasiatic speaking tribes in this region, this result strongly support the view that the migration of Austroasiatic people to Indian subcontinent was mainly male mediated [21]. On the other hand, some of the West Eurasian-specific haplogroups viz. R2, U1, U7 and X were also present at low frequency. Among the M lineages, M3, M5, M35, M39, M40 and M45 haplogroups were present in all the three studied populations, while in R and U lineages, only R6 and U2 were observed among all populations under study (Table 2 and Table S3). It is remarkable that each and every tribal population show a unique partial overlapping package of different haplogroups: approximately 38% individuals of Bharia showed haplogroups, frequent among Munda speakers (M6, M45, R7 and R8); along with other haplogroups (e.g. M33 and M53). M33 has been reported mainly from northeastern and Gujarat states, while M53 is frequent in Chhattisgarh state of India [22]. Haplogroups M2, M3, M5, M30, R5 and U2 were frequent in Bhils while M5, M31, M40, R6, U2, N5 and X were substantially present in Sahariya population (Table 2). Notably, in contrast to other studied and neighboring populations, Sahariya is showing a high frequency of South Asian-specific haplogroup N5 (24%) and West Asian-specific haplogroup X (7%), while, none of the populations of this state showing even a single individual belonging to these haplogroups (Table 2). To make demographic inferences about these particular haplogroups more continental wise data coverage and complete mtDNA sequences are needed.

**Figure 4:**
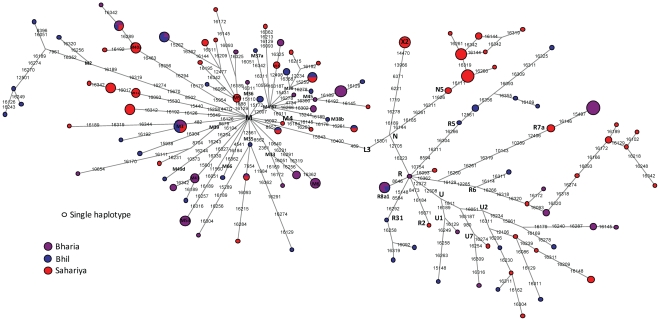
The Median joining network of 209 mtDNAs belonging to three studied populations. Each sample represented on the diagram has been sequenced for the HVS-I region and genotyped for the coding region mutations that are indicated. Circle sizes are proportional to the number of mtDNAs with that haplotype. 16182C, 16183C and 16519 polymorphisms were omitted. Suffixes A, C, G and T indicate transversions.

A phylogenetic network based on HVS-I and coding region mutations, using the MJ NETWORK approach [18] was constructed to determine the genetic relationship between haplotypes of studied populations (Fig. 4). Out of 110 haplotypes detected only two are shared among all the studied populations, while two are shared between Bharia-Bhil, three are shared between Bhil-Sahariya and only one haplotype is shared between Bharia-Sahariya (Fig. 4). We have also noticed novel coding region mutations (e.g. 8557, 15172 and 15649 in the background of haplogroup M which are likely representing at least three new lineages of M, but due to absence of complete mtDNA information we have listed them as M* paragroup (Fig. 4). A maximum parsimony rooted tree was also constructed on the basis of 20 complete mtDNA sequences (Fig. S1). Addition of these novel sequences has increased the resolution of South Asian mtDNA tree and also enabled us to define and refine several South Asian specific subclades. We defined a new branch of haplogroups M35(d), M53(c), R5a2b5, R6a1b and N5(b), while we refined M2a1a3, M5a2a2, M41c, M49d, M30f, M37a3, M66a, R6a1a, R7a1 and R8a1a1c by adding our novel complete sequences (Fig. S1).

### Principal component analysis

**Figure 5:**
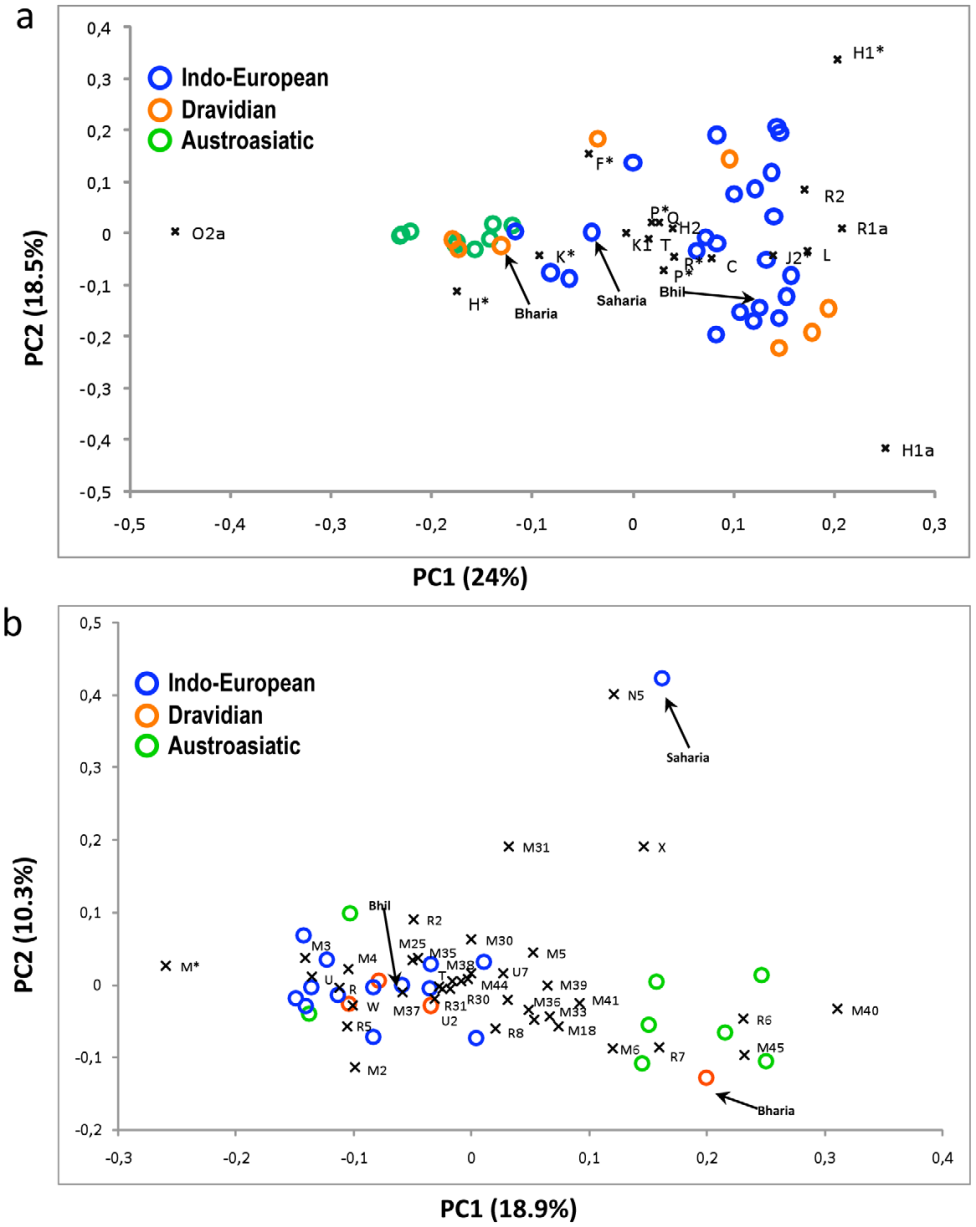
Principal Component Analysis (PCA). PCA plots constructed on the basis of (a) Y SNP haplogroup frequencies and (b) mtDNA haplogroup frequencies. Comparative data were taken from the Indo-European and Austroasiatic populations from the adjoining regions and states of Madhya Pradesh [3], [5], [7], [9], [10], [13], [22], [28].

PCA plots (Fig. 5) were constructed based on the haplogroup frequencies for both Y-chromosome and mtDNA haplogroups in comparison with the other IE, DRA and AA populations from the adjoining regions and states. In Y-chromosomal PCA plot (Fig. 5a) PC1 differentiates AA and non-AA clusters due to haplogroup O2a, which stretches the plot and populations due to its overwhelming presence among AA and few non AA populations. Bharia was found to cluster with AA group, Sahariya positions between these two AA and non AA clusters, while, Bhil shows a closer affinity with IE populations. In mtDNA PCA plot (Fig. 5b), due to high frequency of frequent haplogroups of AA, Bharia clustered with AA populations, Sahariya singled out from the rest over PC2, because of haplogroup N5 which was exclusively present among them. Bhil remains in another cluster along with non-AA and few AA populations (Fig. 5). Thus, as apparent from our mtDNA and Y-chromosome analysis, Bhil tribe has closer affinity with other IE speaking populations even though living nearby the AA groups (Fig. 1). This is possibly due to the different social boundaries among the Indo-Europeans irrespective of their co-inhabitation with other groups and the uprising urbanization.

### Autosomal analysis

**Figure 6:**
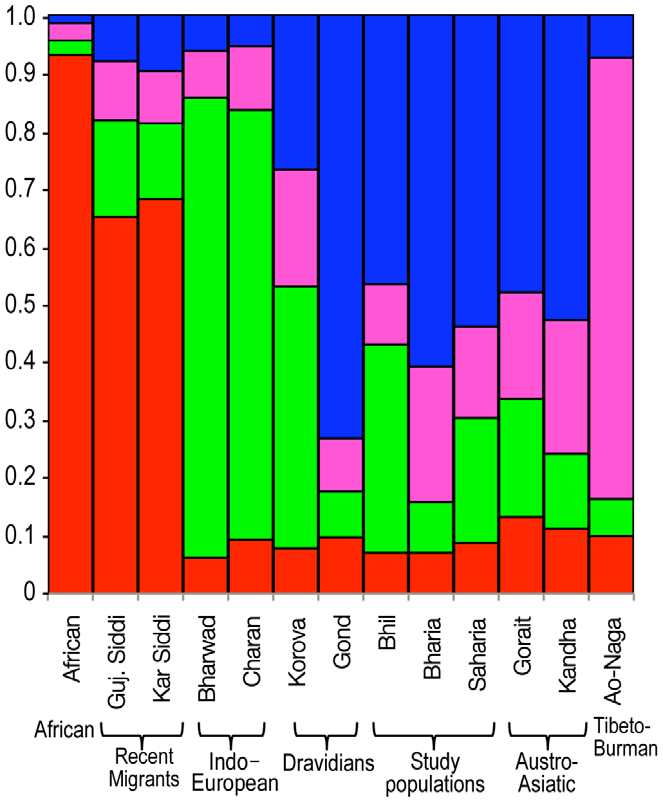
Ancestry sharing analysis by STRUCTURE. STRUCTURE analysis of the populations typed for 48 AIMs. Individuals are represented as thin vertical lines partitioned into segments corresponding to the inferred membership in K = 4 genetic clusters as indicated by the colors.

For STRUCTURE analysis, we selected 13 populations including Africans (Table S4). For a wide coverage and robustness of our analysis based upon 48 AIM's, we have also genotyped Siddi and their neighboring populations to see whether the analysis yield the same ancestry proportion as shown based upon >650 K SNPs [8]. We also included a northeastern Indian population; Ao-Naga as a proxy of East and Southeast Asia. Pattern of population structure of Siddi population obtained at K = 4 was consistent with our previous study and showed largely similar ancestry component sharing (Fig. 6). The distribution of components among Bhil, Bharia and Sahariya showed a similar genetic structuring as haploid DNA results discussed previously. These results advocate that though these populations are quite closely related genetically, it is possible to detect population substructure at such a low marker density.

Overall, our extensive haploid and diploid genetic markers genotyping confirms an Austroasiatic affinity of Bharia in contrast to its language affiliation. The geneflow from AA to Bharia is ancient and might have occurred much before the differentiation of language groups. The presence of rare mtDNA haplogroups (N5 and X) in Sahariya tribe is noteworthy and needs further continent wise high resolution analysis. The genetic signature of the Bhils reflects the typical IE group owing to the strict social customs that is rigid to any incoming genepool. Therefore, the linguistic tag doesn't universally follow a genetic id and in region such as in Central India, it is a prime example of the discordance between languages and genes.

## Materials and Methods

### Blood samples collection and DNA isolation

About 5 ml of blood sample was collected from 65 individuals from Bharias of Chhindwara region, 49 individuals of Bhils from Sehore district and 95 individuals of Sahariya from Shivpuri district of Madhya Pradesh (Fig. 1) with the informed written consent of all the donors. This project has been approved by the Institutional Ethical Committee (IEC) of Centre for Cellular and Molecular Biology (CCMB). The individuals were healthy and unrelated as recognized by the personal interrogation. Each blood sample was subjected to DNA isolation using standard protocol [23]. Thus, the present study comprised 209 mtDNA and 153 Y-chromosome from the three tribes. The details of the samples are given in Table S1 and Fig. 1.

### Y-chromosome analysis

A total of 153 DNA samples (Bharia: 50, Bhil: 30 and Sahariya: 73) were analyzed for the Y-chromosomal biallelic markers. The analysis was done using 16 biallelic markers viz. M9, M45, M82, M69, M175, M173, M17, M11, M172, M89, apt, M124, M70, M130, M147 and M95. The cycling conditions with an initial denaturation of 5 min at 95°C, followed by 35 cycles of 30 sec at 94°C, 30 sec at the primer-specific annealing temperature (52–60)°C, and extension at 72°C for 2 min followed by final extension of 7 min at 72°C were performed. The amplicons were then sequenced using BigDye™ (Applied Biosystems, Foster City, USA) Terminator cycle sequencing kit in ABI Prism 3730 DNA Analyzer following manufacturer's protocol.

A battery of 17 Y-STR loci (DYS456, DYS3891, DYS390, DYS389II, DYS458, DYS19, DYS385a/b, DYS393, DYS391, DYS439, DYS635, DYS392, DYS437, DYS438, DYS448 and Y GATA H4) consisting in the AmpFℓSTR® Y-filer™ PCR amplification Kit (Applied Biosystems, Foster City, USA) was genotyped. This multiplex polymerase chain reaction was carried with 0.5 ng template using manufacturer's instructions. The amplification conditions followed are (1) 95°C for 10 min, (2) 28 cycles; 94°C for 1 min, 55°C for 1 min, 72°C for 1 min, (3) 60°C for 45 min, and (4) 25°C hold. ABI 3730 DNA Analyser (Applied Biosystems, Foster City, USA) was used to analyse the amplicons and the GeneMapper V4.0 software program (Applied Biosystems, Foster City, USA) was used to analyse the raw data.

### Mitochondrial DNA analysis

The hypervariable segment-I (HVS-I) and selected coding region were amplified using 10 ng of the DNA template, with 10 pM of each primer, 200 µM dNTPs, 1.5 mM MgCl_2_ and 1 U of *Taq* DNA polymerase. 35 cycles of reaction was carried out with 30 seconds (sec) denaturation at 94°C, 30 sec. annealing at 58°C and 2 minutes (min) extension at 72°C. The PCR conditions and time duration was modified for different sets of primers whenever necessary. Sequencing of the PCR amplicons was then performed using BigDye™ Terminator cycle sequencing kit (Applied Biosystems, Foster City, USA) in ABI Prism 3730 DNA Analyzer following manufacturer's protocol. The mtDNA sequences obtained were assembled and aligned with the revised Cambridge Reference Sequence (rCRS) [24], using Auto-Assembler ver. 1.4 (Applied Biosystems, Foster City, USA). On the basis of the variations observed, haplogroups were assigned to each sample following the data set from Global human mtDNA phylogenetic tree [20]. Complete mtDNA genome sequences generated in this study were submitted to GenBank (accession number GU480001- GU480020).

### Autosomal Analysis

We genotyped 213 samples from 13 different populations for 50 Ancestry Informative markers (AIM's) using the Sequenom iPLEX assay where two markers failed to give results in all the individuals, therefore our final analysis was based upon 48 AIM's. The detailed information about these markers is published elsewhere [25], and the details about populations are given in Table S4.

### Statistical analysis

Fragment sizes of Y-STRs, were determined using the GeneMapper® Analysis Software v4.0 and allele designations were based on comparison with allelic ladders included in the Yfiler™ kit. Out of 17 loci obtained, two DYS385 loci were excluded from the current analyses because they could not be distinguished using the typing method employed. DYS 389I (DYS 389cd) was subtracted from DYS389II and re-named DYS389ab. Thus, all the analysis linked with Y-STRs data were carried out with 15 loci. A median-joining network, resolved with the MP algorithm, was constructed using the Network package (version 4.5.0.2) (www.fluxus-engineering.com). The age of M95-O2a, M82-H1a and M17-R1a was estimated from microsatellite variation within the haplogroup using the method described by Zhivotovsky et al. [19] and updated in Sengupta et al. [10]. Moreover, Bharia founder was identified based on Network based founder analysis with Munda speakers. The age of these founders was estimated from the ρ statistic (the mean number of mutations from the assumed root of each and every founder), using a 25-year generation time and the TD statistic, assuming a mutation rate of 6.9×10^−4^ [19] based on variation at 15 common Y-STRs loci (Table S2).

Principal component analysis (PCA) was performed using POPSTR, kindly provided by H. Harpending. We ran STRUCTURE [26] for the full data set (48 SNPs and 213 individuals) from *K* = 2 to *K* = 8 (10 runs at each *K*) and selected the *K* (K = 4) that maximizes the posterior probability of the data, as explained by the developers [27]. All structure runs performed 40,000 iterations after a burn-in of 50,000, following the default settings i.e. the admixture model, and assumed that allele frequencies were correlated.

## Supporting Information

Figure S1 **The most parsimonious tree complete mtDNA sequences observed in the three studied populations.** This tree was redrawn manually from the output of median joining/reduced network obtained using NETWORK program (http://www.fluxus-engineering.com). 16182C, 16183C and 16519 polymorphisms were omitted. Suffixes A, C, G, and T indicate transversions. Synonymous (s) and non-synonymous (ns) mutations are distinguished. Recurrent mutations are underlined.
(DOC)

Table S1 **The details about the studied populations including sampling region, total population, linguistic affiliation and occupation.**
(DOC)

Table S2 **The Y-STR profile of M95-O2a, M82-H1a andM17-R1a haplogroup from Bharia, Bhil and Sahariya tribes.**
(DOC)

Table S3 **Population wise HVS-I and coding region mutation list observed among studied populations.**
(XLS)

Table S4 **The detailed information of ancestry component distribution among populations analysed for 48 AIM autosomal markers.** Guj.-Gujarat, Kar.-Karnataka, ANI-Ancestral North Indian, ASI-Ancestral South Indian, SEA-Southeast Asia, *n*- number of samples.
(DOC)

## References

[1] Chaubey G (2010). The demographic history of India: A perspective based on genetic evidence.. http://hdl.handle.net/10062/15240.

[2] Census of India [http://www.censusindia.net/]

[3] Metspalu M, Kivisild T, Metspalu E, Parik J, Hudjashov G (2004). Most of the extant mtDNA boundaries in south and southwest Asia were likely shaped during the initial settlement of Eurasia by anatomically modern humans.. BMC Genet.

[4] Thangaraj K, Chaubey G, Singh VK, Vanniarajan A, Thanseem I (2006). In situ origin of deep rooting lineages of mitochondrial Macrohaplogroup ‘M’ in India.. BMC Genomics.

[5] Kivisild T, Rootsi S, Metspalu M, Mastana S, Kaldma K (2003). The genetic heritage of the earliest settlers persists both in Indian tribal and caste populations.. Am J Hum Genet.

[6] Reich D, Thangaraj K, Patterson N, Price AL, Singh L (2009). Reconstructing Indian population history.. Nature.

[7] Chaubey G, Metspalu M, Choi Y, Mägi R, Romero IG (2011). Population Genetic Structure in Indian Austroasiatic speakers: The Role of Landscape Barriers and Sex-specific Admixture.. Mol Biol Evol.

[8] Shah AM, Tamang R, Moorjani P, Rani DS, Govindaraj P (2011). Indian siddis: African descendants with Indian admixture.. Am J Hum Genet.

[9] Sahoo S, Singh A, Himabindu G, Banerjee J, Sitalaximi T (2006). A prehistory of Indian Y chromosomes: evaluating demic diffusion scenarios.. Proc Natl Acad Sci U S A.

[10] Sengupta S, Zhivotovsky LA, King R, Mehdi SQ, Edmonds CA (2006). Polarity and temporality of high-resolution y-chromosome distributions in India identify both indigenous and exogenous expansions and reveal minor genetic influence of Central Asian pastoralists.. Am J Hum Genet.

[11] Field J, Petraglia M, Lahr M (2007). The southern dispersal hypothesis and the South Asian archaeological record: examination of dispersal routes through GIS analysis.. BMC Biol.

[12] Patnaik R, Chauhan P (2009). India at the cross-roads of human evolution.. J Biosci.

[13] Kumar V, Reddy ANS, Babu JP, Rao TN, Langstieh BT (2007). Y-chromosome evidence suggests a common paternal heritage of Austro-Asiatic populations.. BMC Evol Biol.

[14] Wells RS, Yuldasheva N, Ruzibakiev R, Underhill PA, Evseeva I (2001). The Eurasian heartland: a continental perspective on Y-chromosome diversity.. Proc Natl Acad Sci USA.

[15] Cordaux R, Aunger R, Bentley G, Nasidze I, Sirajuddin SM (2004). Independent origins of Indian caste and tribal paternal lineages.. Curr Biol.

[16] Chaubey G, Metspalu M, Kivisild T, Villems R (2007). Peopling of South Asia: investigating the caste-tribe continuum in India.. Bioessays.

[17] Thangaraj K, Naidu BP, Crivellaro F, Tamang R, Upadhyay S (2010). The influence of natural barriers in shaping the genetic structure of maharashtra populations.. PloS one.

[18] Bandelt HJ, Forster P, Rohl A (1999). Median-joining networks for inferring intraspecific phylogenies.. Mol Biol Evol.

[19] Zhivotovsky LA, Underhill PA, Cinnioglu C, Kayser M, Morar B (2004). The effective mutation rate at Y-chromosome short tandem repeats, with application to human population-divergence time.. Am J Hum Genet.

[20] van Oven M, Kayser M (2009). Updated comprehensive phylogenetic tree of global human mitochondrial DNA variation.. Hum Mutat.

[21] Chaubey G, Karmin M, Metspalu E, Metspalu M, Selvi-Rani D (2008). Phylogeography of mtDNA haplogroup R7 in the Indian peninsula.. BMC Evol Biol.

[22] Chandrasekar A, Kumar S, Sreenath J, Sarkar BN, Urade BP (2009). Updating phylogeny of mitochondrial DNA macrohaplogroup m in India: dispersal of modern human in South Asian corridor.. PloS one.

[23] Thangaraj K, Joshi MB, Reddy AG, Gupta NJ, Chakravarty B (2002). CAG repeat expansion in the androgen receptor gene is not associated with male infertility in Indian populations.. J Androl.

[24] Andrews RM, Kubacka I, Chinnery PF, Lightowlers RN, Turnbull DM (1999). Reanalysis and revision of the Cambridge reference sequence for human mitochondrial DNA.. Nat Genet.

[25] Dhandapany PS, Sadayappan S, Xue Y, Powell GT, Rani DS (2009). A common MYBPC3 (cardiac myosin binding protein C) variant associated with cardiomyopathies in South Asia.. Nat Genet.

[26] Hubisz MJ, Falush D, Stephens M, Pritchard JK (2009). Inferring weak population structure with the assistance of sample group information.. Molecular ecology resources.

[27] Rosenberg NA, Pritchard JK, Weber JL, Cann HM, Kidd KK (2002). Genetic structure of human populations.. Science.

[28] Chaubey G, Metspalu M, Karmin M, Thangaraj K, Rootsi S (2008). Language shift by indigenous population: a model genetic study in South Asia.. International Journal of Human Genetics.

