# Supplementary material for: Genetic Affinities of the Central Indian Tribal Populations
Source: PLoS One. 2012 Feb 29;7(2):e32546. doi: 10.1371/journal.pone.0032546 (PMC3290590; doi:10.1371/journal.pone.0032546)
Supplement: Table S1 — The details about the studied populations including sampling region, total population, linguistic affiliation and occupation. (DOC) [file pone.0032546.s002.doc]

| **Tribe** | **District** | **Total population**  **(Census 2001)** | **Linguistics** | **Occupation** | **Total** |
| --- | --- | --- | --- | --- | --- |
| Bharia | Chhindwara | 20,890 | Dravidian | Farmers/ Labours | 65 |
| Bhil | Sehore | 46,415 | Indo-European | Farmers / Labours | 49 |
| Sahariya | Shivpuri | 1,39,124 | Indo-European | Forest wood collectors/ Labours | 95 |
