# Supplementary material for: Genetic Affinities of the Central Indian Tribal Populations
Source: PLoS One. 2012 Feb 29;7(2):e32546. doi: 10.1371/journal.pone.0032546 (PMC3290590; doi:10.1371/journal.pone.0032546)
Supplement: Supplementary file 2 [file pone.0032546.s001.doc]

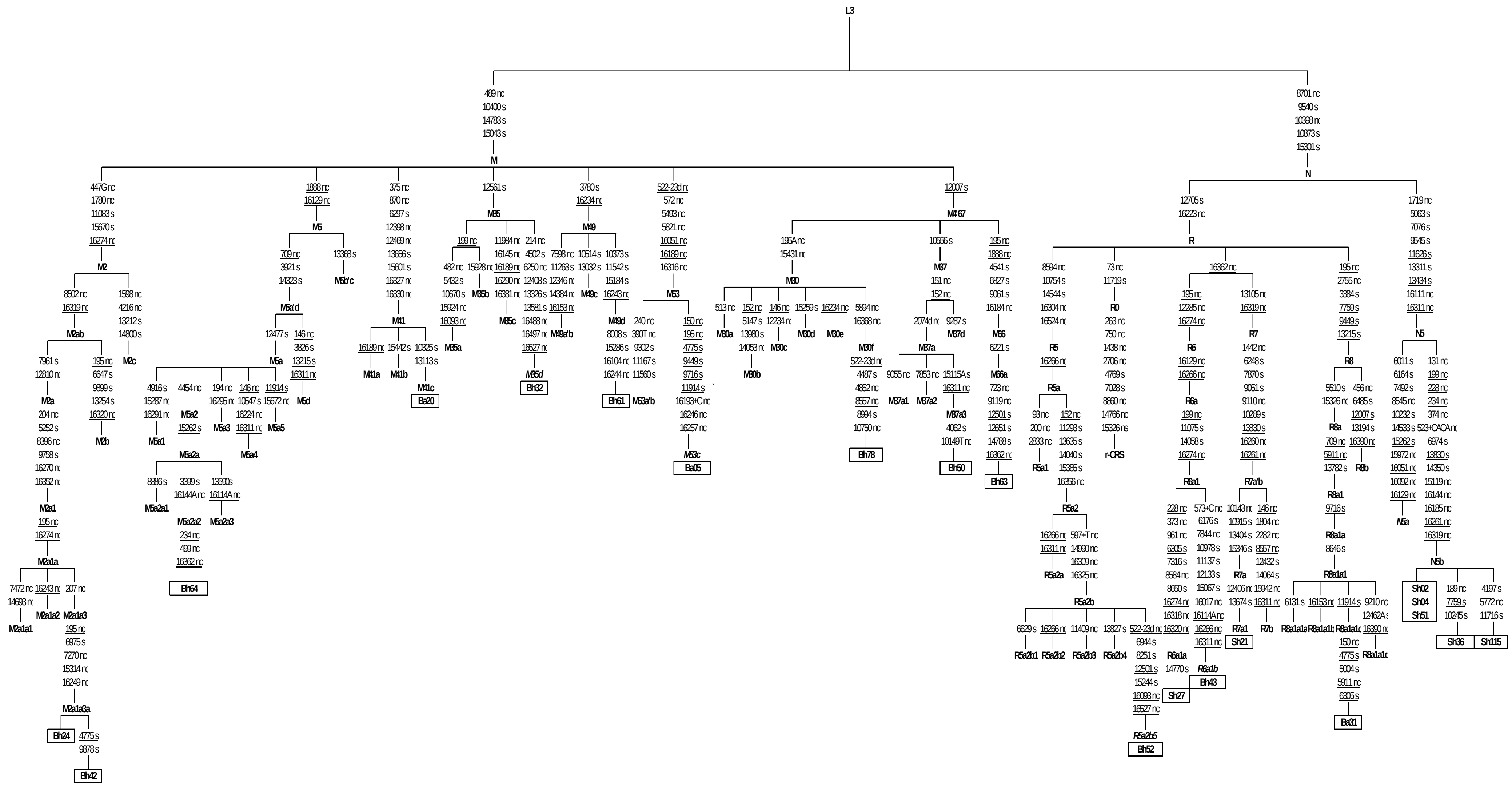

L3
M
N
R
N5
M2
M5
M35
M49
M53
M4'67
M30
M37
M66
R5
R0
R6
R7
R8
M2a
M2b
M2c
M5a
M41
M5a2
R5a
R8a
N5b
Bh24
Bh42
Bh64
Ba20
Bh32
Bh61
Ba05
Bh78
Bh50
Bh63
Bh52
Sh27
Bh43
Sh21
Ba31
Sh02
Sh04
Sh51
Sh36
Sh115
