# Supplementary material for: Genetic Affinities of the Central Indian Tribal Populations
Source: PLoS One. 2012 Feb 29;7(2):e32546. doi: 10.1371/journal.pone.0032546 (PMC3290590; doi:10.1371/journal.pone.0032546)
Supplement: Supplementary file 3 [file pone.0032546.s003.doc]

Population	HG	Sample File	DYS19	DYS385a	DYS385b	DYS389ab	DYS389cd	DYS390	DYS391	DYS392	DYS393	DYS437	DYS438	DYS439	DYS448	DYS456	DYS458	DYS635	GATA_H4
Bhil	H1	Bi 12	15	15	16	16	12	22	11	13	11	14	9	11	18	15	18	20	12
Bhil	H1	Bi 13	15	15	16	16	12	22	10	11	11	14	9	11	19	16	18	20	12
Bhil	H1	Bi 14	15	15	16	16	12	22	11	13	11	14	9	11	18	16	17	20	11
Bhil	H1	Bi 15	15	15	16	16	12	22	10	11	11	14	9	11	19	16	18	20	12
Bhil	H1	Bi 17	15	15	16	16	12	22	11	13	11	14	9	11	18	15	18	21	12
Bhil	H1	Bi 19	15	15	16	16	12	22	10	11	11	14	9	11	19	16	17	21	12
Bhil	H1	Bi 20	16	15	16	16	12	22	11	13	11	14	9	11	18	16	17	20	11
Bhil	H1	Bi 29	15	15	18	16	13	22	10	11	11	14	9	11	19	15	19	20	12
Bhil	H1	Bi 3	14	16	17	16	13	21	10	11	11	14	9	12	20	17	16	20	13
Bhil	H1	Bi 30	15	16	17	16	13	23	10	11	11	14	9	11	19	15	17	20	11
Bhil	H1	Bi 31	14	16	17	16	13	21	10	11	11	14	9	12	20	17	16	20	13
Bhil	H1	Bi 34	15	13	18	17	12	21	9	10	11	13	10	9	19	14	15	22	12
Bhil	H1	Bi25	14	16	17	16	13	21	10	11	11	14	9	12	20	17	16	20	13
Bhil	H1	Bi-33	14	16	17	16	13	21	10	11	11	14	9	12	20	17	16	20	13
Bhil	H1	Bi-34	15	15	18	16	13	22	10	11	11	14	9	11	19	15	19	20	12
Bharia	O2a	Ba 2	15	14	19	15	13	25	10	13	13	14	11	12	18	17	15	21	11
Bharia	O2a	Ba 29	15	14	18	16	14	25	10	13	14	14	10	12	18	17	16	21	11
Bharia	O2a	Ba 30	15	14	19	16	14	25	10	13	14	14	10	12	18	17	16	21	11
Bharia	O2a	Ba 31	15	15	20	17	13	24	11	13	12	14	11	13	18	17	16	21	11
Bharia	O2a	Ba 33	15	15	20	17	13	23	11	13	13	14	11	13	18	17	16	21	11
Bharia	O2a	Ba 34	15	17	20	16	12	25	10	13	13	14	10	13	19	15	15	22	11
Bharia	O2a	Ba 35	15	14	19	16	14	25	10	13	13	14	10	12	18	17	16	21	11
Bharia	O2a	Ba 36	15	17	20	16	13	25	10	13	13	14	10	12	18	15	15	23	11
Bharia	O2a	Ba 38	15	16	20	16	12	25	10	13	12	14	10	12	18	16	15	23	11
Bharia	O2a	Ba 39	15	15	19	16	13	25	11	13	12	14	11	13	18	17	16	22	11
Bharia	O2a	Ba 41	16	17	18	17	13	25	10	13	13	14	10	13	18	15	15	21	12
Bharia	O2a	Ba 43	15	15	17	16	13	25	11	13	11	14	11	13	18	17	16	22	11
Bharia	O2a	Ba 49	15	16	20	16	12	25	10	13	13	14	10	12	18	16	15	23	11
Bharia	O2a	Ba 8	15	14	19	17	14	25	10	13	13	14	10	12	18	16	16	21	12
Bharia	O2a	Ba12	15	14	19	16	14	25	10	13	13	14	10	12	18	17	16	21	11
Bharia	O2a	Ba13	15	15	20	16	13	25	11	13	12	14	11	11	18	17	17	22	11
Bharia	O2a	Ba16	15	15	20	16	13	25	11	13	12	14	11	12	18	17	16	22	11
Bharia	O2a	Ba17	15	14	19	16	14	25	10	13	13	14	10	12	18	16	16	20	11
Bharia	O2a	Ba20	15	15	19	16	13	25	11	13	11	14	11	13	18	17	16	22	11
Bharia	O2a	Ba24	15	15	19	16	13	25	11	13	12	14	11	12	18	16	16	22	11
Bharia	O2a	Ba28	15	15	18	17	13	25	11	13	13	14	10	12	18	15	16	22	11
Bharia	O2a	Ba5	15	17	19	16	13	25	10	13	13	14	10	13	18	14	16	23	11
Bharia	O2a	Ba7	15	15	19	16	13	25	11	13	12	14	11	13	18	17	16	22	11
Bharia	O2a	Ba8	15	14	19	17	14	25	10	13	13	15	10	12	18	16	16	21	12
Saharia	O2a	S1	15	14	19	16	13	25	10	13	13	14	10	12	18	16	16	22	12
Saharia	O2a	S2	15	15	19	17	13	25	10	13	14	14	10	13	18	15	16	21	11
Saharia	O2a	S35	16	17	20	16	13	25	10	13	13	14	10	11	18	16	15	21	11
Saharia	O2a	S4	15	16	18	16	14	25	10	13	13	14	10	12	18	16	16	21	11
Saharia	O2a	S44	15	17	22	18	12	25	10	13	12	14	10	11	18	16	15	23	12
Saharia	O2a	S54	15	13	18	16	13	24	10	13	14	14	10	12	18	15	16	22	11
Saharia	O2a	S57	15	17	20	17	12	25	10	13	12	14	10	11	18	17	15	22	12
Saharia	O2a	S58	15	11	14	17	14	25	10	11	12	14	11	10	21	15	17	23	13
Saharia	O2a	S59	15	14	19	16	13	24	10	13	14	14	10	13	18	16	16	22	11
Saharia	O2a	S61	14	17	21	17	12	24	10	13	12	14	10	11	18	16	16	24	12
Saharia	O2a	S62	15	14	19	16	13	25	10	13	15	14	10	12	18	16	16	22	11
Saharia	O2a	S65	15	14	19	16	13	25	10	13	13	14	10	13	18	16	16	21	11
Saharia	O2a	S68	14	14	21	16	14	24	10	13	13	14	10	12	18	16	16	21	11
Saharia	O2a	S69	15	17	21	16	13	25	10	13	12	14	10	11	18	17	15	23	11
Saharia	O2a	S70	15	17	20	17	12	25	10	13	12	14	10	12	18	17	15	23	12
Saharia	O2a	S72	15	17	20	17	12	25	10	13	12	14	10	12	18	17	15	22	12
Saharia	O2a	S9	15	14	18	17	13	25	10	13	12	14	11	13	21	17	16	22	11
Saharia	R1a	S115	15	11	14	18	14	24	10	11	12	14	11	10	20	15	16	23	12
Saharia	R1a	S24	15	11	14	18	14	24	10	11	12	14	11	10	20	15	16	23	12
Saharia	R1a	S25	15	14	19	17	14	24	10	11	12	14	11	10	20	15	16	23	13
Saharia	R1a	S28	16	11	15	18	13	25	10	11	12	14	11	10	20	15	18	24	12
Saharia	R1a	S3	15	10	15	17	13	25	10	11	12	14	11	9	20	15	16	23	13
Saharia	R1a	S34	15	11	14	18	14	24	10	11	12	14	11	10	20	15	16	24	12
Saharia	R1a	S37	15	14	17	18	14	24	10	12	13	14	11	10	20	16	16	23	12
Saharia	R1a	S39	18	14	17	15	15	28	12	14	13	17	?	13	22	17	17	?	?
Saharia	R1a	S42	16	11	14	17	13	25	10	11	12	14	11	10	20	15	16	23	12
Saharia	R1a	S5	15	12	21	16	13	23	10	10	11	16	10	11	18	16	?	22	12
Saharia	R1a	S50	15	11	14	17	14	22	11	11	12	14	11	10	20	15	16	23	13
Saharia	R1a	S53	15	11	14	17	14	25	10	11	12	14	11	10	21	15	18	23	13
Saharia	R1a	S6	15	11	14	18	14	24	10	11	12	14	11	10	20	15	16	23	12
