# Supplementary material for: Genetic Affinities of the Central Indian Tribal Populations
Source: PLoS One. 2012 Feb 29;7(2):e32546. doi: 10.1371/journal.pone.0032546 (PMC3290590; doi:10.1371/journal.pone.0032546)
Supplement: Supplementary file 4 [file pone.0032546.s005.doc]

Population	African	ANI	ASI	East/SEA	*n*
African	0.93	0.025	0.015	0.03	11
Guj. Siddi	0.65	0.167	0.081	0.102	25
Kar. Siddi	0.681	0.131	0.098	0.09	25
Bharwad	0.062	0.794	0.063	0.081	10
Charan	0.093	0.742	0.055	0.11	10
Korova	0.078	0.452	0.268	0.202	20
Gond	0.097	0.079	0.732	0.092	17
Bhil	0.07	0.36	0.466	0.104	15
Bharia	0.063	0.079	0.547	0.211	15
Saharia	0.087	0.216	0.539	0.158	15
Gorait	0.132	0.204	0.48	0.184	15
Kandha	0.112	0.129	0.528	0.231	15
Ao-Naga	0.099	0.064	0.075	0.762	20
